# Lack of impact of ipragliflozin on endothelial function in patients with type 2 diabetes: sub-analysis of the PROTECT study

**DOI:** 10.1186/s12933-023-01856-x

**Published:** 2023-05-20

**Authors:** Shinji Kishimoto, Yukihito Higashi, Takumi Imai, Kazuo Eguchi, Kazuo Fukumoto, Hirofumi Tomiyama, Koji Maemura, Atsushi Tanaka, Koichi Node, the PROTECT investigators, Toyoaki Murohara, Masafumi Kitakaze, Yoshihiko Nishio, Teruo Inoue, Mitsuru Ohishi, Kazuomi Kario, Masataka Sata, Michio Shimabukuro, Wataru Shimizu, Hideaki Jinnouchi, Isao Taguchi, Makoto Suzuki, Shinichi Ando, Haruo Kamiya, Tomohiro Sakamoto, Hiroki Teragawa, Mamoru Nanasato, Munehide Matsuhisa, Junya Ako, Yoshimasa Aso, Masaharu Ishihara, Kazuo Kitagawa, Akira Yamashina, Tomoko Ishizu, Yumi Ikehara, Shinichiro Ueda, Ayako Takamori, Hisako Yoshida, Miki Mori, Kaori Yamaguchi, Machiko Asaka, Tetsuya Kaneko, Masashi Sakuma, Shigeru Toyoda, Takahisa Nasuno, Michiya Kageyama, Jojima Teruo, Iijima Toshie, Haruka Kishi, Hirotsugu Yamada, Kenya Kusunose, Daiju Fukuda, Shusuke Yagi, Koji Yamaguchi, Takayuki Ise, Yutaka Kawabata, Akio Kuroda, Yuichi Akasaki, Mihoko Kurano, Satoshi Hoshide, Takahiro Komori, Tomoyuki Kabutoya, Yukiyo Ogata, Yuji Koide, Hiroaki Kawano, Satoshi Ikeda, Satoki Fukae, Seiji Koga, Masato Kajikawa, Tatsuya Maruhashi, Yoshiaki Kubota, Yoshisato Shibata, Nehiro Kuriyama, Ikuko Nakamura, Kanemitsu Hironori, Bonpei Takase, Yuichi Orita, Chikage Oshita, Yuko Uchimura, Ruka Yoshida, Yukihiko Yoshida, Hirohiko Suzuki, Yasuhiro Ogura, Mayuho Maeda, Masaki Takenaka, Takumi Hayashi, Mirai Hirose, Itaru Hisauchi, Toshiaki Kadokami, Ryo Nakamura, Junji Kanda, Kazuo Matsunaga, Masaaki Hoshiga, Koichi Sohmiya, Yumiko Kanzaki, Arihiro Koyosue, Hiroki Uehara, Naoto Miyagi, Toshiya Chinen, Kentaro Nakamura, Chikashi Nago, Suguru Chiba, Sho Hatano, Yoshikatsu Gima, Masami Abe, Masayoshi Ajioka, Hiroshi Asano, Yoshihiro Nakashima, Hiroyuki Osanai, Takahiro Kanbara, Yusuke Sakamoto, Mitsutoshi Oguri, Shiou Ohguchi, Kunihiko Takahara, Kazuhiro Izumi, Kenichiro Yasuda, Akihiro Kudo, Noritaka Machii, Ryota Morimoto, Yasuko Bando, Takahiro Okumura, Toru Kondo, Shin-ichiro Miura, Yuhei Shiga, Joji Mirii, Makoto Sugihara, Tadaaki Arimura, Junko Nakano, Tomohiro Sakamoto, Kazuhisa Kodama, Nobuyuki Ohte, Tomonori Sugiura, Kazuaki Wakami, Yasuhiko Takemoto, Minoru Yoshiyama, Taichi Shuto, Yosuke Okada, Kenichi Tanaka, Satomi Sonoda, Akemi Tokutsu, Takashi Otsuka, Fumi Uemura, Kenji Koikawa, Megumi Miyazaki, Maiko Umikawa, Manabu Narisawa, Machi Furuta, Hiroshi Minami, Masaru Doi, Kazuhiro Sugimoto, Susumu Suzuki, Akira Kurozumi, Kosuke Nishio

**Affiliations:** 1 Department of Regenerative Medicine, Research Institute for Radiation Biology and Medicine (RIRBM), Hiroshima University, 1-2-3 Kasumi, Minami-ku, Hiroshima, 734-8551 Japan; 2 Division of Regeneration and Medicine, Medical Center for Translational and Clinical Research, Hiroshima University Hospital, Hiroshima, Japan; 3 Department of Medical Statistics, Osaka Metropolitan University Graduate School of Medicine, Osaka, Japan; 4 Department of General Internal Medicine, Saitama Red Cross Hospital, Saitama, Japan; 5 Department of Medical Education and General Practice, Osaka Metropolitan University Graduate School of Medicine, Osaka, Japan; 6 Department of Cardiology, Tokyo Medical University, Tokyo, Japan; 7 Department of Cardiovascular Medicine, Nagasaki University Graduate School of Biomedical Sciences, Nagasaki, Japan; 8 Department of Cardiovascular Medicine, Saga University, Saga, Japan

**Keywords:** Ipragliflozin, Sodium-glucose cotransporter-2 inhibitors, Endothelial function, Type 2 diabetes

## Abstract

**Background:**

We assessed the impact of 24 months of treatment with ipragliflozin, a sodium-glucose cotransporter 2 (SGLT2) inhibitor, on endothelial function in patients with type 2 diabetes as a sub-analysis of the PROTECT study.

**Methods:**

In the PROTECT study, patients were randomized to receive either standard antihyperglycemic treatment (control group, n = 241 ) or add-on ipragliflozin treatment (ipragliflozin group, n = 241) in a 1:1 ratio. Among the 482 patients in the PROTECT study, flow-mediated vasodilation (FMD) was assessed in 32 patients in the control group and 26 patients in the ipragliflozin group before and after 24 months of treatment.

**Results:**

HbA1c levels significantly decreased after 24 months of treatment compared to the baseline value in the ipragliflozin group, but not in the control group. However, there was no significant difference between the changes in HbA1c levels in the two groups (7.4 ± 0.8% vs. 7.0 ± 0.9% in the ipragliflozin group and 7.4 ± 0.7% vs. 7.3 ± 0.7% in the control group; P = 0.08). There was no significant difference between FMD values at baseline and after 24 months in both groups (5.2 ± 2.6% vs. 5.2 ± 2.6%, P = 0.98 in the ipragliflozin group; 5.4 ± 2.9% vs. 5.0 ± 3.2%, P = 0.34 in the control group). There was no significant difference in the estimated percentage change in FMD between the two groups (P = 0.77).

**Conclusions:**

Over a 24-month period, the addition of ipragliflozin to standard therapy in patients with type 2 diabetes did not change endothelial function assessed by FMD in the brachial artery.

***Trial registration*:**

Registration Number for Clinical Trial: jRCT1071220089 (https://jrct.niph.go.jp/en-latest-detail/jRCT1071220089).

## Introduction

Sodium glucose cotransporter 2 (SGLT2) inhibitors are oral glucose medicines that lower glucose levels by reducing the renal reabsorption of glucose. Some meta-analyses and large clinical trials have shown that SGLT2 inhibitors reduce cardiovascular events in patients with type 2 diabetes and reduced the rate of hospitalization for heart failure in patients with heart failure [1–10]. Inzucchi et al. [11] showed that changes in hematocrit and hemoglobin levels in the EMPA-REG OUTCOME trial might be major mediators of empagliflozin-induced decreases in the incidence of cardiovascular events. SGLT2 inhibitors induce diuresis and glycosuria to decrease the intravascular volume and lower the cardiac preload and afterload, which consequently increase the cardiac output. These impacts of SGLT2 inhibitors may lead to improvements in endothelial function. Unfortunately, the effect of long-term treatment with SGLT2 inhibitors on endothelial function in patients with type 2 diabetes is still unclear.

Endothelial dysfunction is regarded as the first stage in the etiology of atherosclerosis and plays a significant role in the progression of atherosclerosis, leading to cardiovascular issues [12, 13]. Measurements of flow-mediated vasodilation (FMD) have been widely used as an indication of endothelial function in the brachial artery [14, 15]. Several studies have shown that endothelial dysfunction predicts cardiovascular events [16, 17].

The PROTECT study was a multicenter prospective study designed to test the inhibitory impact of an SGLT2 inhibitor on the development of atherosclerosis based on intima-media thickness over a 24-month follow-up period [18, 19]. FMD of the brachial artery was assessed in some participants. The present study’s purpose, which was a sub-analysis of the PROTECT study, was to determine the impacts of 24 months of treatment using SGLT2 inhibitors on endothelial function as measured by FMD in the brachial artery in patients with type 2 diabetes.

## Methods

### Study design and patients

The rationale and design of the PROTECT study (University Hospital Medical Information Network Center: ID000018440) have already been explained [18]. In brief, the PROTECT study was a multicenter, prospective, randomized, open-label, blinded-endpoint investigator-initiated clinical trial in which 39 Japanese institutions that participated in patients with HbA1c levels of 6.0–10.0% despite conventional treatment with diet, exercise, and/or pharmacological therapy with prescribed diabetic drugs for more than 3 months before randomization were eligible for the study if they were at least 20 years old and had type 2 diabetes. Patients who had taken an SGLT2 inhibitor one month before randomization were excluded. The other exclusion criteria have been described elsewhere [18].

**Fig. 1:**
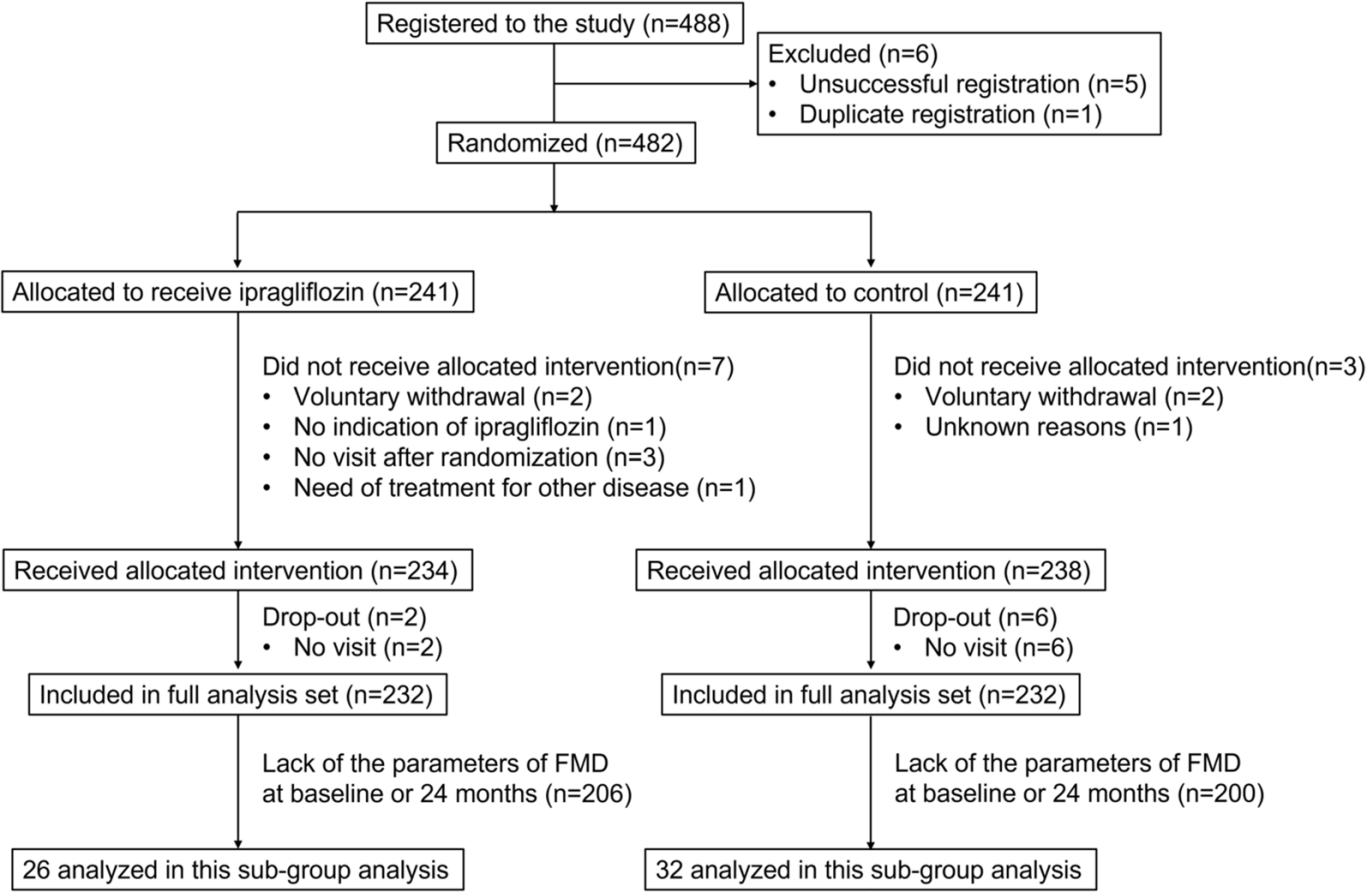
Flow diagram of participants in the PROTECT study

Between September 2015 and June 2018, 488 patients with type 2 diabetes were enrolled, and 482 patients were randomized to receive either standard antihyperglycemic treatment (control group, n = 241) or add-on ipragliflozin treatment (ipragliflozin group, n = 241) at a 1:1 ratio (Fig. 1). The treatment randomization was conducted on the basis of age (< 65 or ≥ 65 years), HbA1c level (< 7% or ≥ 7%), office systolic blood pressure (< 135 or ≥ 135 mm Hg), use of statins, and use of metformin at the time of screening [18]. Patients in the ipragliflozin group were initiated with ipragliflozin at a dose of 50 mg daily. If a personalized goal in accordance with the official recommendations regarding HbA1c levels from the Japan Diabetes Society was not achieved, the dose of ipragliflozin was increased to 100 mg daily, and the background treatment for participants in the control group continued. The background therapy for the participants remained unaltered in both groups throughout the trial, within the acceptable limits of the therapeutic goal. If the personalized goal was not achieved, antihyperglycemic agents other than SGLT2 inhibitors and/or insulin were administered to both groups. However, because pioglitazone has a suppressive effect on the progression of intima-media thickness (IMT) [20], the prescription of pioglitazone or a change in its dose was prohibited during the study. All patients were followed up for 24 months after starting the study protocol.

In the PROTECT study, the primary endpoint in the main analysis was the change in the mean common carotid artery IMT from baseline to 24 months after the start of treatment; the results have already been reported [19]. The main objective of the present sub-analysis of the change in FMD in the brachial artery from baseline to 24 months after the start of treatment was to analyze one of the prespecified secondary endpoints [18]. FMD of the brachial artery was assessed at some participating institutions as an additional examination.

Of the 488 patients, serial FMD measurements were performed in 32 patients in the control group and 26 patients in the ipragliflozin group before and after 24 months of treatment (Fig. 1). In the present study, data from 58 patients from six institutions were examined. The study protocol for this sub-analysis was approved by the Ethics Committee of Saga University Hospital (2022-09-02) and registered (jRCT1071220089). All the individuals provided written informed consent to participate in the study. All methods were performed in accordance with the Declaration of Helsinki and relevant guidelines and regulations in Japan.

### FMD measurement protocol

After overnight fasting, all experiments were conducted in the morning. All of the patients were kept in the supine position in a calm, dark, air-conditioned room with a constant temperature of 22 ℃–25 ℃ throughout the study. A 23-gauge polyethylene catheter was placed in the left deep antecubital vein to collect blood samples. Endothelium-dependent FMD was assessed based on the vascular response to reactive hyperemia in the brachial artery. Observers were blinded to the examinations.

FMD was measured using the same ultrasound device designed for FMD measurements and the same protocol in all institutions. A high-resolution linear artery transducer was coupled to computer-assisted analysis software (UNEXEF18G, UNEX Co, Nagoya, Japan), which uses an automated edge detection system for the measurement of brachial artery diameter [21]. A blood pressure cuff was placed around the forearm. The brachial artery was scanned longitudinally 5–10 cm above the elbow. When the clearest B-mode image of the anterior and posterior intimal interfaces between the lumen and the vessel wall was obtained, the transducer was held at the same point throughout the scan using a special probe holder (UNEX Co.) to ensure image consistency. The depth and gain were set to optimize the images of the arterial lumen wall interface. When the tracking gate was placed on the intima, the artery diameter was automatically tracked, and the waveform of the diameter changed over the cardiac cycle was displayed in real-time using the FMD mode of the tracking system. This allowed the ultrasound images to be optimized at the start of the scan, and the transducer position to be adjusted immediately for optimal tracking performance throughout the scan. Pulsed Doppler flow was assessed at baseline and during peak hyperemic flow, which was confirmed to occur within 15 s after cuff deflation. The blood flow velocity was calculated from color Doppler data and displayed as a waveform in real time. Baseline longitudinal images of the artery were acquired for 30 s, and the blood pressure cuff was inflated to 50 mmHg above the systolic pressure for 5 min. Longitudinal images of the artery were recorded continuously for 5 min after cuff deflation. Pulsed Doppler velocity signals were obtained for 20 s at baseline and 10 s immediately after cuff deflation. Changes in the brachial artery diameter were immediately expressed as percentage changes relative to the vessel diameter before cuff inflation. FMD was automatically calculated as the percentage change in the peak vessel diameter from the baseline value. The percentage of FMD [(peak diameter–baseline diameter)/baseline diameter] was used for the analysis. The blood flow volume was calculated by multiplying the Doppler flow velocity (corrected for the angle) with the heart rate and vessel cross-sectional area (r2). Reactive hyperemia was calculated as the maximum percentage increase in flow after cuff deflation compared with the baseline flow. In our laboratory, the inter- and intra-coefficients of variation for the brachial artery diameter were 1.6% and 1.4%, respectively, in our laboratory [22].

### Statistical analysis

Results are presented as means ± SD. Statistical significance was defined as a probability value < 0.05, and all stated probability values were two-sided. The chi-square test was used to compare categorical variables and an unpaired Student’s t-test was used to compare the mean values of continuous variables between the groups. A paired Student’s t-test was used to assess the differences in the mean values of continuous variables between baseline and 24 months. A linear regression model was used to estimate the changes in FMD over time by treatment (control vs. ipragliflozin group). To estimate the group differences in the percentage changes in FMD, the models included treatment, age, sex, and baseline FMD. Data were processed using R 4.0.1 (R Foundation for Statistical Computing, Vienna, Austria).

## Results

### Baseline clinical characteristics

The baseline clinical characteristics of all patients are shown in Table 1, along with the impacts of each therapy on baseline variables in the ipragliflozin and control groups. Of the 58 patients, 39 (67.2%) were men, and 19 (32.8%) were women. Nine patients (15.5%) were current smokers, 37 (63.8%) had hypertension, 28 (48.3%) had dyslipidemia, 22 (37.9%) had atherosclerotic cardiovascular disease, and 4 (6.9%) had a previous stroke.

**Table 1:** Clinical Characteristics of the Subjects

Variables	All (n = 58)	Control group (n = 32)			Ipragliflozin group (n = 26)		
		0 month	24 months	P value	0 month	24 months	P value
Age, yr	65.1 ± 10.4	66.3 ± 10.4			63.5 ± 10.4		
Male, n (%)	39 (67.2)	22 (68.8)			17 (65.4)		
Body mass index, kg/m^2^	28.3 ± 5.0	28.1 ± 5.9	27.9 ± 5.5	*0.30*	28.4 ± 3.8	26.7 ± 3.8*	*< 0.01*
Systolic blood pressure, mmHg	132 ± 14	134 ± 14	133 ± 13	*0.80*	130 ± 14	128 ± 13	*0.56*
Diastolic blood pressure, mmHg	77 ± 14	76 ± 14	73 ± 10	*0.12*	76 ± 14	74 ± 13	*0.40*
Heart rate, bpm	70 ± 15	67 ± 12	71 ± 13	*0.05*	73 ± 17	74 ± 14	*0.75*
Total cholesterol, mmol/L	4.57 ± 0.55	4.61 ± 0.55	4.32 ± 0.87	*0.01*	4.52 ± 0.67	4.63 ± 0.90**	*0.39*
HDL cholesterol, mmol/L	1.25 ± 0.33	1.35 ± 0.35	1.31 ± 0.26	*0.23*	1.12 ± 0.26*	1.20 ± 0.20	*0.03*
LDL cholesterol, mmol/L	2.62 ± 0.61	2.62 ± 0.69	2.40 ± 0.55	*0.04*	2.62 ± 0.52	2.68 ± 0.73**	*0.45*
Glucose, mmol/L	8.25 ± 1.31	8.3 ± 1.87	7.85 ± 1.67	*0.21*	8.19 ± 1.77	7.24 ± 1.35	*< 0.01*
HbA1c, %	7.4 ± 0.7	7.4 ± 0.7	7.3 ± 0.7	*0.27*	7.4 ± 0.8	7.0 ± 0.9	*< 0.01*
eGFR, mL/min/1.73m^2^	71.7 ± 18.1	73.7 ± 20.6	74.7 ± 21.2	*0.49*	69.3 ± 14.6	69.1 ± 15.5**	*0.88*
Current smoker, n (%)	9 (15.5)	7 (21.9)			2 (7.7)		
Medical history, n (%)							
Hypertension	37 (63.8)	21 (65.6)			16 (61.5)		
Dyslipidemia	28 (48.3)	14 (43.8)			14 (53.8)		
Atherosclerotic cardiovascular disease	22 (37.9)	10 (31.2)			12 (46.2)		
Previous stroke	4 (6.9)	2 (6.2)			2 (7.7)		
Heart failure	8 (13.8)	4 (12.5)			4 (15.4)		
Medication, n (%)							
ACE inhibitors	9 (15.5)	5 (15.6)	5 (15.6)	*1.00*	4 (15.4)	4 (15.4)	*1.00*
ARBs	36 (62.1)	22 (68.8)	22 (68.8)	*1.00*	14 (53.8)	16 (61.5)	*0.57*
Calcium channel blockers	43 (74.1)	26 (81.2)	26 (81.2)	*1.00*	17 (65.4)	17 (65.4)	*1.00*
Beta-blockers	24 (41.4)	12 (37.5)	14 (43.8)	*0.61*	12 (46.2)	12 (46.2)	*1.00*
Statins	38 (65.5)	21 (65.6)	21 (65.6)	*1.00*	17 (65.4)	17 (65.4)	*1.00*
Antiplatelet drugs	32 (41.6)	13 (41.9)	12 (38.7)	*0.80*	9 (34.6)	9 (34.6)	*1.00*
Insulin	1 (1.7)	0 (0.0)	0 (0.0)	*1.00*	1 (3.8)	2 (7.7)	*0.55*
Metformin	24 (41.4)	15 (46.9)	16 (50.0)	*0.80*	9 (34.6)	10 (38.5)	*0.77*
Sulfonylurea	10 (17.2)	8 (25.0)	7 (21.9)	*0.77*	2 (7.7)	1 (3.8)	*0.55*
Thiazolidinedione	0 (0.0)	0 (0.0)	2 (6.2)	*0.09*	0 (0.0)	0 (0.0)	*N/A*
DPP-4 inhibitors	37 (63.8)	19 (59.4)	19 (59.4)	*1.00*	18 (69.2)	16 (61.5)	*0.57*
GLP-1 receptor agonists	0 (0.0)	0 (0.0)	0 (0.0)	*N/A*	0 (0.0)	1 (3.8)	*0.24*
FMD, %	*5.3 ± 2.7*	*5.4 ± 2.9*	*5.0 ± 3.2*	*0.34*	*5.2 ± 2.6*	*5.2 ± 2.6*	*0.96*

Results are presented as means ± SD for continuous variables and percentages for categorical variables

*HDL *high-density lipoprotein, *LDL* low-density lipoprotein, *HbA1c* hemoglobin A1c, *eGFR* estimated glomerular filtration rate, *ACE* angiotensin-converting enzyme,  *ARB* angiotensin II receptor blocker, *DPP-4* dipeptidyl peptidase-4, *GLP-1* Glucagon-like peptide-1,  *N/A* not applicable, *FMD* flow-mediated vasodilation

*P < 0.01 vs. control group

**P < 0.05 vs. control group

The mean fasting plasma glucose level was 8.25 ± 1.31 mmol/L and mean HbA1c was 7.4 ± 0.7%. The mean value of FMD was 5.3 ± 2.7%. None of the variables, except high-density lipoprotein (HDL) cholesterol, showed significant differences between the two groups.

#### Changes in clinical characteristics after 24 months

In the ipragliflozin group, body mass index was significantly decreased after 24 months (28.4 ± 3.8 vs. 26.7 ± 3.8 kg/m^2^; P < 0.01) and HDL was significantly increased after 24 months (1.12 ± 0.26 vs. 1.20 ± 0.20 mmol/L; P = 0.03). In the control group, total cholesterol and low-density lipoprotein (LDL) cholesterol were significantly decreased after 24 months (4.61 ± 0.55 vs. 4.32 ± 0.87 mmol/L for total cholesterol and 2.62 ± 0.69 vs. 2.40 ± 0.55 mmol/L for LDL; P = 0.01 and P = 0.04, respectively). No significant differences were observed in any other variables after 24 months in either group. The total cholesterol and LDL cholesterol levels after 24 months were significantly higher in the ipragliflozin group than in the control group. The body mass index and estimated glomerular filtration rate after 24 months were significantly lower in the ipragliflozin group than in the control group.

### Glycemic control

The baseline HbA1c and fasting plasma glucose levels were comparable between the two groups. The HbA1c level significantly decreased after 24 months of treatment compared to the baseline value in the ipragliflozin group (7.4 ± 0.8% vs. 7.0 ± 0.9%; P < 0.01), but not in the control group (7.4 ± 0.7% vs. 7.3 ± 0.7%; P = 0.27). However, there was no significant difference between changes in HbA1c levels in the two groups (7.4 ± 0.8% vs. 7.0% ± 0.9% in the ipragliflozin group and 7.4 ± 0.7% vs. 7.3 ± 0.7% in the control group; P = 0.08 Table 1). There was no significant difference in the fasting plasma glucose levels between the two groups during the study period.

### Endothelial function

**Fig. 2:**
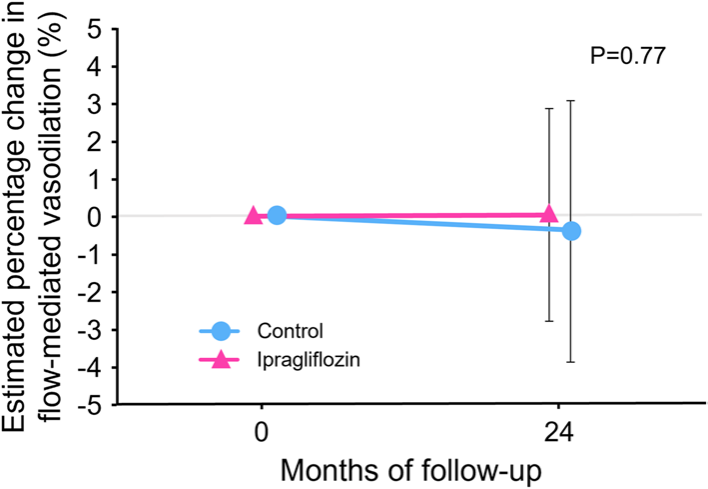
Changes in estimated percentage change in flow-mediated vasodilation (FMD) in the ipragliflozin group and the control group. The mixed-effects model included treatment, age, sex, and FMD at baseline

Figure 2 shows the impacts of glycemic management on FMD after 24 months of treatment in the ipragliflozin and control groups. Baseline FMD values were comparable between the two groups. There was no significant difference between FMD values at baseline and after 24 months in either group (5.2 ± 2.6% vs. 5.2 ± 2.6%, P = 0.98 in the ipragliflozin group and 5.4 ± 2.9% vs. 5.0 ± 3.2%, P = 0.34 in the control group). There was no significant difference in the estimated percentage change in FMD between the two groups (P = 0.77).

## Discussion

The present study demonstrated that adding ipragliflozin to standard therapy for 24 months in patients with type 2 diabetes did not change endothelial function as measured by FMD in the conduit brachial artery.

### Impact of SGLT2 inhibitors on FMD

The short-term impacts of SGLT2 inhibitors on the vascular function in patients with type 2 diabetes are controversially reported [23–25]. The DEFENCE study, a randomized, open-label, blinded-endpoint, parallel-group, comparative clinical trial, showed that dapagliflozin add-on therapy with metformin for four months improved FMD in patients with type 2 diabetes [23]. Sposito et al. [24] showed that 12 weeks of dapagliflozin add-on therapy to metformin improved FMD in patients with type 2 diabetes. Conversely, Zainordin et al. [25] showed that 12 weeks of dapagliflozin add-on therapy to metformin and insulin did not alter FMD, and that there was no significant difference in FMD between the control and dapagliflozin groups. Sposito et al. [26] showed that 16-week treatment with empagliflozin did not alter FMD. The EMBLEM trial, a multicenter, randomized, placebo-controlled, double-blind trial, showed that empagliflozin administered for 24 weeks did not alter the reactive hyperemia peripheral arterial tonometry index as an indication of endothelial function in patients with type 2 diabetes [27]. These findings suggest that the impacts of short-term dapagliflozin administration on endothelial function remain unclear. In addition, the long-term impacts of dapagliflozin on endothelial function remain unclear. The present study showed that 24 months of ipragliflozin treatment did not alter FMD in patients with type 2 diabetes.

### Impact of SGLT2 inhibitors on endothelial function

Several clinical trials have shown that SGLT2 inhibitors can prevent cardiovascular events in patients with type 2 diabetes and heart failure [4–10]. In addition, a meta-analysis revealed that SGLT2 inhibitors prevent major adverse cardiovascular events in patients with type 2 diabetes [1–3]. Suzuki et al. [28] showed that the cardiovascular risk associated with individual SGLT2 inhibitors in patients with type 2 diabetes was comparable, based on large-scale real-world data. These findings suggest that SGLT2 inhibitors exert anti-atherosclerotic impacts. However, the exact mechanisms underlying the anti-atherosclerotic impacts of SGLT2 inhibitors remain unclear. It has been postulated that one of the anti-atherosclerotic impacts of SGLT2 inhibitors is improvement of endothelial function. Several investigators have shown that SGLT2 inhibitors improve nitric oxide (NO) bioavailability by inhibiting inflammatory reactions and decreasing oxidative stress [29–31]. Salim et al. [32] showed that ipragliflozin improves endothelial function in diabetic mice through enhancement of phosphorylation of Akt and endothelial NO synthase and reduced urinary excretion of 8-hydroxy-2’-deoxyguanosine as an index of oxidative stress. D’Onofrio et al. [33] showed that treatment with SGLT2 inhibitors improved plaque stability and decreased 2-year outcomes in diabetic patients, potentially by modulating the SGLT2/SIRT6 pathway. Ripoll et al. [34] showed that the beneficial effects of dapagliflozin on endothelial barrier integrity are mediated through a critical downstream link involving the apolipoprotein M/sphingosine-1-phosphate pathway. However, in the present study, ipragliflozin did not alter FMD in patients with type 2 diabetes.

### Study limitations

This study has some limitations. First, the number of subjects in the present study, as a sub-analysis of the PROTECT trial, was relatively small. Because FMD was an optional measurement in the PROTECT trial, there was an insufficient sample size for power calculation, and the analysis may have been underpowered. However, the results of analysis of the PROTECT trial, a multicenter, prospective, randomized, open-label, and blinded-endpoint investigator-initiated clinical trial, provide valuable information that may help us to understand the impact of ipragliflozin on endothelial function in patients with type 2 diabetes. Further studies with a larger number of participants are required to validate the long-term impacts of SGLT2 inhibitors on endothelial function in patients with type 2 diabetes. Second, LDL cholesterol levels in the control group after 24 months were significantly lower than those in the ipragliflozin group after 24 months, although there was no change in the use of dyslipidemia-improving drugs, including statins, ezetimibe, fibrates, and eicosapentaenoic acid, in either group. Lowering LDL cholesterol levels improves vascular endothelial function. The lower LDL cholesterol level in the control group at 24 months may have been one reason for the lack of a significant difference in FMD between the two groups. However, there was no significant difference in FMD between the two groups after adjusting for LDL cholesterol.

## Conclusion

Over a 24-month period, the addition of ipragliflozin to standard therapy in patients with type 2 diabetes did not change the endothelial function assessed by FMD in the brachial artery.

## Data Availability

The data are available upon reasonable request from researchers who submit a detailed proposal outlining their intended use of the data and after approval by the principal investigators and steering committee of the PROTECT study. Inquiries must be addressed to the corresponding author (or the study secretariat: substudy_protect@clin-med.org).

## Abbreviations

FMD: Flow-mediated vasodilation
HDL: High-density lipoprotein
IMT: Intima-media thickness
LDL: Low-density lipoprotein
NO: Nitric oxide
SGLT2: Sodium-glucose cotransporter 2
